# Anévrisme de l'artère fémorale superficielle lié à une infection à salmonelle

**DOI:** 10.11604/pamj.2015.22.218.7804

**Published:** 2015-11-10

**Authors:** Melek Ben Mrad, Mohammed Ben Hammamia, Rim Miri, Bilel Derbel, Mohammed Ali Koubaa, Jalel Ziadi, Adel Khayati

**Affiliations:** 1 Service de Chirurgie Cardio-Vasculaire, Hôpital La Rabta, Tunis, Tunisie

**Keywords:** Anévrisme mycotique, artère fémorale superficielle, salmonelle, mycotic aneurysm, superficial femoral artery, salmonella

## Abstract

Les anévrismes infectieux de l'axe fémoro-poplité sont rares et d’évolution silencieuse. Ils surviennent surtout chez les sujets immunodéprimés. Leur traitement fait appel souvent à l'exclusion chirurgicale associée ou non à un geste de revascularisation. Nous rapportons le cas d'un patient opéré pour un anévrisme de l'artère fémorale superficielle, lié à une infection à salmonelle, il a eu une résection de l'anévrysme et une revascularisation par greffe veineuse. Ce patient a été repris par la suite à deux reprise pour rupture du greffon veineux. Pour cette raison, nous étions contraints à une explantation du pontage et une ligature des 2 bouts de l'artère sans revascularisation. Malgré l'absence de revascularisation, l’évolution a été favorable sous couverture d'une une antibiothérapie adaptée. En cas d'anévrysme infectieux de l'axe fémoro-poplité par infection à salmonelle, le rétablissement de la continuité artérielle, même avec un greffon veineux, peut exposer au risque de rupture artérielle.

## Introduction

Les anévrismes infectieux de l'axe fémoro-poplité sont rares et d’évolution silencieuse. Ils surviennent volontiers chez les personnes immunodéprimées ou révèlent de l'iatrogénie. Leur traitement fait appel souvent à l'exclusion chirurgicale associée ou non à un geste de revascularisation. Nous rapportons le cas d'un patient opéré à plusieurs reprises pour un anévrisme de l'artère fémorale superficielle (AFS) lié à une infection à Salmonelle.

## Patient et observation

**Figure 1:**
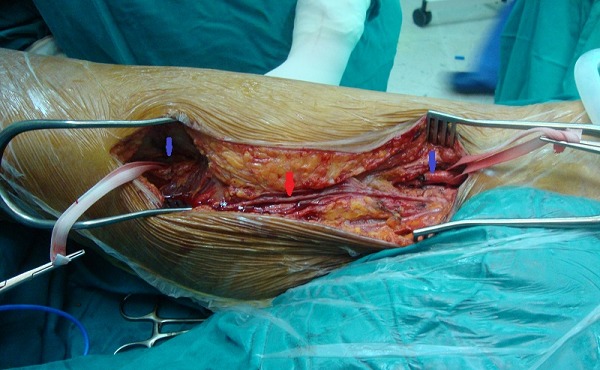
Cliché peropératoire montrant le contrôle premier de l'artère fémorale superficielle de part et d'autre de l'anévrysme (flèches bleus) et la dissection de la veine saphène interne (flèche rouge)

**Figure 2:**
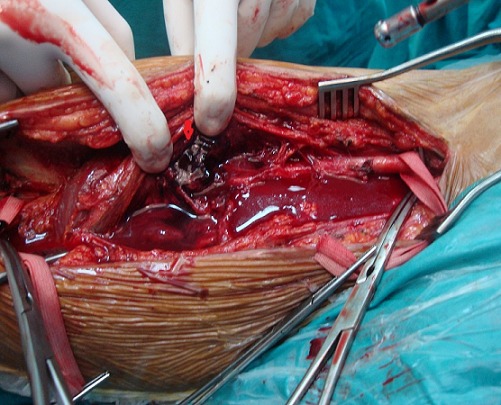
Cliché peropératoire montant un faux anévrysme infecté de l'artère fémorale superficielle (flèche)

**Figure 3:**
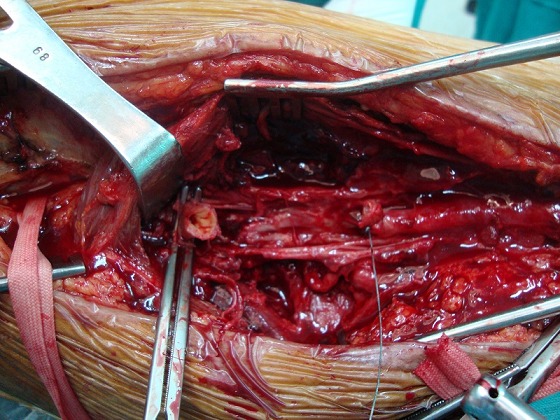
Cliché peropératoire montant la résection totale de l'anévrysme infecté de l'artère fémorale superficielle

**Figure 4:**
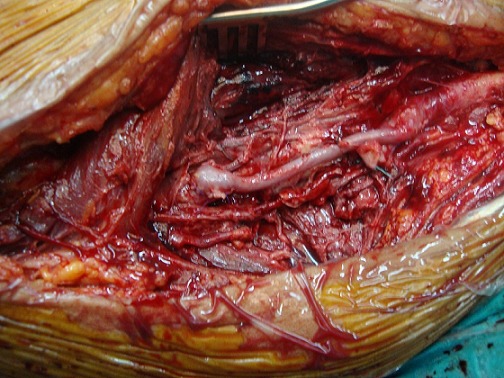
Pontage veineux termino-terminal en veine saphène inverse

**Figure 5:**
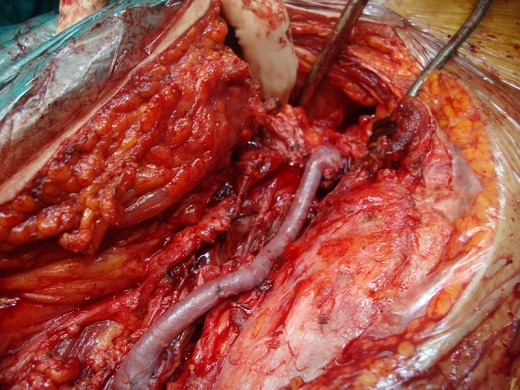
Le deuxième pontage veineux qui a été réalisé après la rupture du premier. Sa longueur est plus importante que le premier avec un trajet plus externe

Le patient M.G âgé de 49 ans hypertendu et suivi pour une hépatite B chronique sous interféron, a été hospitalisé dans notre service pour une masse battante localisée au niveau de la face interne de la cuisse. L'angioscanner a montré un anévrisme non compliqué du tiers moyen de l'AFS faisant 5 cm de diamètre. Nous avons opté pour une cure chirurgicale de cet anévrisme. L'intervention s'est déroulée sous anesthésie générale, après control de l'AFS de part et d'autre de l'anévrysme et prélèvement de la veine saphène (Figure 1), l'exploration a objectivé un anévrysme infecté et rompu de l'AFS (Figure 2), la décision était de faire un débridement élargi avec résection de tout le tissu artériel infecté (Figure 3), le rétablissement de la continuité artérielle a été réalisé par un pontage veineux termino-terminal en veine saphène homolatérale prélevé au même site opératoire (Figure 4). Les suites opératoires ont été marquées par la survenue d'un saignement provenant du site opératoire 2 jours après l'intervention. Le patient a été repris, l'exploration chirurgicale a objectivé une ulcération au niveau du pontage veineux qui est à l'origine du saignement associée à une infection des parties molles. Nous avons réalisé une excision du tissu infecté et une confection d'un nouveau pontage veineux au même site opératoire (Figure 5). Les suites opératoires ont été marquées encore une fois par un saignement du site opératoire 4 jours après la 2^ème^ intervention. Nous avons repris le patient une 2^ème^ fois, le saignement provenait cette fois ci de l'anastomose proximale que nous avons réparée. Quatre jours plus tard, le patient a présenté un saignement du site opératoire. Notre attitude était radicale cette fois et nous avons réalisé une explantation du pontage veineux avec ligature des 2 bouts de l'AFS associée à un débridement chirurgical élargi. L’évolution sur le plan local était favorable; le membre inférieur gardait une perfusion acceptable par la collatéralité bien développée et n’était pas en ischémie, mais sur le plan général, le patient est devenu fébrile avec une altération de l’état général. Les prélèvements bactériologiques per opératoires ont permis d'isoler un "Salmonelle SPP". Nous avons mis le patient sous antibiothérapie adaptée à base d'Ampicilline et ciprofloxacine pendant 40 jours. L’échographie cardiaque trans-thoracique ainsi que celle trans-osophagienne sont revenues normales. L’évolution ultérieure a été marquée par une détersion totale des stigmates d'infections et une nette amélioration sur le plan général avec obtention de l'apyrexie. Cependant, le patient a gardé des claudications intermittentes avec un périmètre de marche à 200 mètres.

## Discussion

Les lésions artérielles anévrismales sont souvent liées au processus arthéroscléreux. Les anévrismes mycotiques sont rares et restent l'apanage des sujets immunodéprimés atteints du virus d'immunodéficience humaine ou de cancers [1]. Notre patient avait une hépatite B chronique sous interféron pouvant expliquer la survenue d'une telle infection. Les germes les plus fréquemment responsables des anévrismes mycotiques sont représentés par le Staphylocoque aureus, l'Escherichia Coli et le Salmonelle Spp [2]. Ce dernier est le plus souvent associé aux anévrismes thoraciques et abdominaux et sa localisation au niveau des artères fémorales reste exceptionnelle [3–5]. Avant l’ère des antibiotiques, les anévrismes infectieux, qualifiés de «mycotiques» en 1885 par William Osler [1], avaient le plus souvent été rapportés sous la forme d'une localisation aortique au cours d'endocardites à streptocoque. Pour cette raison, la recherche d'une localisation secondaire en particulier cardiaque au cours d'un anévrisme infectieux périphérique est indispensable. Nous avons pratiqué une échographie trans-‘sophagienne pour notre patient qui n'a pas montré de végétations endocardiques. Le traitement de ces anévrismes fait appel souvent à la chirurgie. La résection artérielle constitue le traitement de référence mais la rétablissement de la continuité artérielle n'est pas toujours indiqué et représente un sujet de controverse dans la prise en charge des anévrismes infectieux à cause d'un taux élevé de rupture et de réinfection secondaire variant de 10% à 35% [6]. Les veines saphènes internes et les veines fémorales superficielles constituent des greffons adéquats dans ces indications malgré un taux de réinfection important [6]. Cependant, certains auteurs préfèrent l'utilisation de greffon artériel pour le rétablissement de la continuité après la mise à plat. Klonaris et al [7] ont rapporté des résultats satisfaisants après utilisation de l'artère iliaque interne comme greffon. D'autres auteurs préfèrent l'utilisation de la veine saphène interne in situ pour le traitement de ces anévrismes [8]. Nous pensons que surseoir à un geste de revascularisation et se limiter à une simple ligature de part et d'autre de l'AFS après la résection est une option qui ne doit pas être exclue. Toutefois, cette option est associée à un taux d'ischémie et d'amputation majeure important de l'ordre de 34% [9], mais son grand avantage c'est qu'elle permet d’éviter les infections de nono et les saignements post opératoires nécessitant des reprises itératives et pouvant même mettre en jeu le pronostic vital du patient. Dans le cas de notre patient, la ligature des 2 bouts de l'AFS n'a pas entrainé une perte de membre, toutefois le patient a développé une ischémie chronique avec une claudication intermittente mais qui n'est pas très gênante. Le traitement endovasculaire des anévrismes infectieux a été rapporté comme alternative à la chirurgie conventionnelle. Certes cette approche présente un taux de morbi-mortalité moindre, mais les infections des endoprothèses ont été rapportées et elles ont une évolution souvent fatale. Pour cette raison, ce traitement reste actuellement réservé pour les patients en mauvais état général ou inopérables [10]. Qu'il soit chirurgical ou endovasculaire, le traitement des anévrismes de l'AFS liés à une infection à salmonelle doit être associé à une antibiothérapie adaptée pour une durée prolongée sur laquelle il n'existe pas de consensus mais qui devrait être d'au moins 6 semaines pour certains auteurs [2]. Les céphalosporines de 3^ème^ génération demeurent le traitement de choix actuellement en raison de l’émergence de résistances à l'ampicilline dans 65% des cas et à la ciprofloxacine dans 41% des cas [4].

## Conclusion

Les anévrismes infectieux de l'AFS sont l'apanage des sujets immunodéprimés. Leur traitement se base sur la résection chirurgicale et l'antibiothérapie adaptée. Le rétablissement de la continuité artérielle, même avec un greffon veineux, peut exposer au risque de rupture artérielle secondaire. La résection simple avec débridement peut être une option viable.
